# Does social pension buy improved mental health and mortality outcomes for senior citizens? Evidence from South Africa's 2008 pension reform

**DOI:** 10.1016/j.pmedr.2022.102026

**Published:** 2022-10-17

**Authors:** Cyprian M. Mostert, Diana Mackay, Alex Awiti, Manasi Kumar, Zul Merali

**Affiliations:** Aga Khan University, Brain and Mind Institute, 3rd Parklands Avenue, Nairobi, Kenya

**Keywords:** Pension, Mental health, Elderly health, South Africa

## Abstract

In South Africa, men were traditionally eligible to receive government pensions at 65 years. However, that eligibility criterion was changed in 2008 to allow men to receive a pension payout at 60 years. This study is designed to quantify the impact of the 2008 pension reform on mental health outcomes (depression and traumatic stress) and deaths among 60-year-old men from disadvantaged households without advanced education. This analysis used secondary data issued by Statistic South Africa- General Household Survey. Men who reported earning a pension at 60 years from 2008 to 2014 were exposed to the 2008 pension reform and thus were classified as the treatment group. The 60-year-old men during 2002–2007 were ineligible to earn the pension, therefore considered the control group. We then used a Two-stage Least Squared Model (2SLS) to quantify the impact of the 2008 pension reform on healthcare utilization, depression cases, traumatic stress cases, and deaths among 60-year-old men. The model shows that the 2008 pension reform improved healthcare utilization by 3 % in the cohorts of men who benefitted from the 2008 reform. The 2008 pension reform averted depression cases, traumatic stress cases, and deaths among 60-year-old men by 3 %, 4 %, and 5 %, respectively. The impact of the 2008 pension reform in averting deaths among 60-year-old men was higher in urban regions than rural regions. We concluded that the 2008 pension reform successfully bought improved mental health outcomes and prevented depression, traumatic stress, and deaths among 60-year-old men.

## Highlights

• The 2008 pension reform in South Africa improved access to healthcare.
• The 2008 pension reform improved mental health and 60-year-old deaths.
• The impact of the 2008 pension reform in averting 60-year-old deaths is higher in urban regions than rural regions.

## 1 Introduction

One question that remains open for investigation in developing countries is whether social pension succeeds in buying the mental health outcomes of senior citizens (Riumallo-Herl and Aguila, 2019, Hu et al., 2019), especially in the male population group. It is documented that men have higher health risks than women, which justifies early pension provision (Amari and Behnezhad, 2020). However, few developing countries can afford to pay social pensions early, and the impact of this fund on the elderly population's mental health remains unknown.

So far, most studies look at the impact of social pension on living arrangements and intergeneration health outcomes (Hamoudi and Thomas, 2014, Chepngeno-Langat et al., 2019, Herrmann et al., 2021), neglecting the impact of social pension on health outcomes of 60-year-old men. According to the recent review article (Lloyd-Sherlock and Amoakoh-Coleman, 2020), the impact of social pensions on health outcomes of 60-year-old men remains relatively unknown in the sub-Saharan region, despite the popularity of social pensions.

**Fig. 1:**
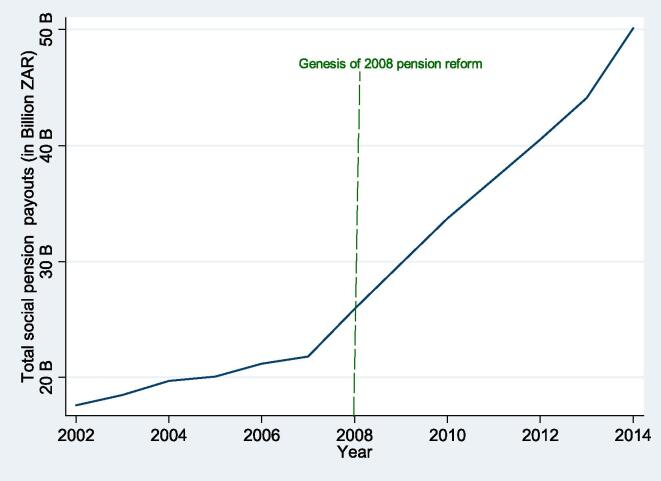
Evolution of pension pay-out in South Africa. Source: Own elaboration with data from SASSA 2002–2014.

**Fig. 2:**
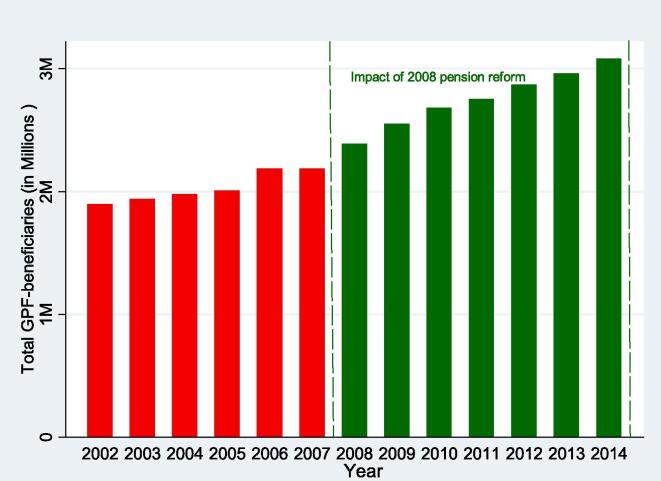
Evolution of pension beneficiaries. Source: Own elaboration with data from SASSA 2002–2014.

South Africa is one of the countries in the sub-Saharan region with extensive social pension coverage, which expanded between 2002 and 2014 (See Fig. 1, Fig. 2 in the Appendix). In 2014, over 3 million senior citizens benefitted from the social pension system. It was reported that the lifetime prevalence rate of mental ill-health – (depression & traumatic stress) in South Africa was 2.3 percent and 4.9 percent, respectively (Swain et al., 2017, Peltzer and Phaswana-Mafuya, 2013). These figures are lower than the global average (De Leo and Giannotti, 2021) – characterized by low pension coverage for the elderly population. There is a possibility that the expanding social pension coverage in South Africa is responsible for improving depression & traumatic stress. This paper is designed to assess such an intersection.

In China, a social pension is reported to improve the mental wellbeing of elderly citizens by relieving depression and stress (Wang and Zheng, 2021). Others show that social pension enhances food availability and contributes to the dietary diversity of elderly citizens (Waidler and Devereux, 2019). These two key factors improve the health outcomes of pension beneficiaries. Hence scholars have suggested that lack of pension provision worsens elderly deaths and violates senior citizens' rights (Ataguba et al., 2021, Brinda et al., 2016). On the other hand, expanding pension provision and early retirement improved senior citizens' mental health and mortality outcomes (Pak, 2021, Sewdas et al., 2020, Oshio and Kan, 2017, McIntyre et al., 2016). However, other authors reject this view, arguing that expanding social pension provision does not improve elderly mortalities (Philipp, 2019). So far, no study in the sub-Saharan region has assessed the intersection between social pension expansion, elderly mental health outcomes, and deaths among 60-year-old men (Lloyd-Sherlock and Amoakoh-Coleman, 2020). The current paper estimates whether social pension expansion concurrently averts mental ill-health (depression and traumatic stress) and deaths among 60-year-old men.

This inquiry is pertinent considering that there are growing calls internationally for governments to consider increasing the pension age starting after 65 years (OECD, 2019, Komp, 2018, Hagemann and Scherger, 2016, Steenkamp, 2017). It is not yet known if delaying age eligibility for social pension provision compromises the health outcomes of senior citizens in the Sub-Saharan region (Pilipiec et al., 2021). This paper seeks to contribute to this literature by demonstrating the power of early pension intervention and expansion on male senior citizens' mental health and mortality outcomes by using South Africa as a case.

## 2 Social pension and South African men

Men in South Africa were traditionally eligible to receive social pensions at 65 years. However, instead of following the international trends, the South African government implemented a 2008 pension reform which changed the age-eligible criterion and allowed men to receive a social pension payout at 60 years–5 years earlier than the general norm (Ralston et al., 2015) (See Fig. 1, Fig. 2 in the Appendix).

The current study seeks to answer the following questions (1) What impacts does the 2008 pension reform exert on male senior citizen (60-year-old) healthcare utilization? (2) What impacts does the 2008 pension reform exert on male senior citizen (60-year-old) depression and traumatic stress cases? (3) What impacts does the 2008 pension reform exert on deaths among 60-year-old men? Lastly, are there regional-based differences in the impact of the 2008 pension reform on 60-year-old deaths? This paper explores findings that demonstrate the potential of the social pension to improve elderly mental health and mortality outcomes in Africa.

## 3 Materials and methods

We used a recently released database, the General Household Survey (GHS), provided by Statistic South Africa from 2002 to 2014. The GHS is an annual survey of approximately 120,000 individuals from more than 20,000 households (Mostert and Vall, 2020, Mostert, 2021, Mostert, 2021, Mostert, 2021). These anonymized databases aim to provide information on changing trends in South African families' composition (Mostert and Vall, 2020, Mostert, 2021, Mostert, 2021, Mostert, 2021). The GHS sampling procedure involves explicit stratification by province and urban and non-urban areas within each province. Household units are drawn under this stratification. For each household unit, individual characteristics are presented, including age, gender, educational outcomes, health outcomes, income levels, social pension beneficiaries, the father's living status: alive or deceased, and other general socioeconomic status variables. Household units are drawn under this stratification (Mostert and Vall, 2020, Mostert, 2021, Mostert, 2021, Mostert, 2021).

The GHS structure questions for a binary response (Mostert and Vall, 2020, Mostert, 2021, Mostert, 2021, Mostert, 2021). For example, senior individuals in the household are asked if elderly members benefit from a social pension grant. The general reply is either yes, the member earns a social pension grant, or no the member does not earn a pension. For health outcomes- the GHS asked if the 60-year-old man was still alive in the household. The general reply is either yes, the man is still alive, or no the man is deceased. Also, the GHS asked if the man had utilized the healthcare facility. The general reply is either yes, the man has used the healthcare facility, or no the man has not utilized the healthcare facility.

The GHS asked if the man had experienced depressive episodes as part of the mental health outcomes assessment. The general reply is either yes, the man has experience depression, or no the man has not experienced any depression. Furthermore, the GHS asked if the man had gone through traumatic stress. The general reply is either yes, the man has experienced traumatic stress, or no the man has not experienced any traumatic stress.

We use these binary variables to quantify the changes in the counts of healthcare utilization, depression cases, traumatic stress cases, and deaths among 60-year-old men in individuals who benefited from the 2008 pension reform versus those who did not benefit from the reform.

**Fig. 3:**
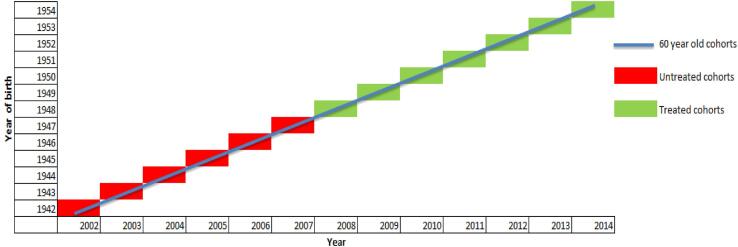
Definition of treated and control groups according to the instrument (year of birth or cohort) for the current health outcomes analysis. Source: Own elaboration according to the South African Social Security Agency (SASSA) pension reform.

We linked the annual datasets of GHS from 2002 to 2014 to create pooled cross-section for the current analysis, like the strategy done by previous studies in South Africa (Mostert and Vall, 2020, Mostert, 2021, Mostert, 2021, Mostert, 2021). Such pooled cross-section assisted us in understanding the impact of the 2008 pension reform on men's health outcomes. We then monitor the changes in the counts of healthcare utilization, depression cases, traumatic stress cases, and 60-year-old deaths in cohorts treated with the 2008 pension reform (60-year cohorts born from 1948 to 1954) (2008–2014). We then compare these outcomes to cohorts not treated with the 2008 pension reform (60-year cohorts born from 1942 to 1947) (2002–2007) (See Fig. 3 in the Appendix). Ethical approval was exempted.

High household income may influence the two groups' mental health and mortality outcomes. To avoid this confounding factor -we analyzed these outcomes in disadvantaged households earning less than US $ 36,567 per year. The outcomes we analyzed are dummy variables reported by the head of the family when asked about the health outcomes of these elderly cohorts: visited the healthcare facility, experienced depression, experienced traumatic stress, and 60-year-old deaths. The blue line in Fig. 3 of the Appendix represents all the 60-year cohorts considered in the current analysis. The cohorts shaded in red refer to controls (because they have not received the benefit of the 2008 pension reform), while those shaded in green are the treated cohorts of the 2008 pension reform.

The GHS also includes information on households who benefit from the 2008 pension reform. However, we did not want to directly compare families who benefit from the pension to those not benefitting as these two groups of households can be different in many additional dimensions (for example, income from relatives, education background, or information barriers), which can influence the exogenous aspect of the model and inflate the possible outcomes. Hence, we did not use an OLS model (we have provided OLS results see the Appendix Table 7, Table 8, Table 9). Instead, we opted for a Two-stage Least Squared Model (2SLS) – like other health policy studies done in South Africa (Mostert and Vall, 2020, Mostert, 2021, Mostert, 2021, Mostert, 2021, Mostert, 2022). The 2SLS models are more robust to biases than other models. They are still preferable in most cases when analyzing studies where the direction of the causal effect is theoretically clear (Collischon and Eberl, 2020)- similar to our current estimation. In this analysis, we instrument the receipt of the 2008 pension with the birth cohort as presented below.

(1) $$Y_{i}^{a}=\alpha_{1}+\beta_{1}\;2008Pension\partial +\psi RaceFE+\xi \mathrm{Pr}ovinceFE+℘YearFE+ƛEducationFE+\sigma Work$$

$$2008Pension=\alpha_{2}+\beta_{2}Treat_{i}^{a}+\psi RaceFE+\xi \mathrm{Pr}ovinceFE+℘YearFE+ƛEducationFE+\sigma Work$$

In the first equation, Y is one of the outcomes for individual i at age ɑ (60 years old), and “∼2008Pension ” is the predicted benefit from the 2008 pension reform. The regression includes race fixed effects (which capture the different racial groups in South Africa); province fixed effects (including nine provinces in South Africa); calendar year fixed effects (which are equivalent to having cohort fixed effects), and a dummy for work (which control take-up of odd jobs to survive). Furthermore, we also include control (fixed effects) for the head of household's educational level to proxy for the household socioeconomic characteristics in line with other cash transfer papers in South Africa (Mostert and Vall, 2020). We omitted households who did not provide answers on health outcomes.

In the second equation (which corresponds to the first stage regression of the 2SLS), the 2008 pension reform is estimated as a function of the treatment dummy variable, which identifies the cohorts exposed to the 2008 pension reform (as depicted in Fig. 3 of the Appendix). Therefore, when one looks at the health outcomes at age 60, it is evident in Fig. 3 of the Appendix that cohorts born between 1942 and 1947 were not affected by the 2008 pension reform, thus constituting the control group. Meanwhile, cohorts born between 1948 and 1954 were affected by the 2008 pension reform and constitute the treatment group. We also control for the head of household's educational level as proxies for household socioeconomic status, which may influence the outcome variables.

The first stage regression also controls the race, province, work, and year fixed effects. Thus, the year (or cohort) fixed effects account for any trend in the outcome variable across 60-year cohorts, and the province fixed effects control for any baseline (time-invariant) difference in the outcome variables across provinces (Mostert and Vall, 2020). In all model estimations, one needs two assumptions to be fulfilled: first, the instrument must be relevant in explaining the probability of being treated, and this will be corroborated by the F-test of the first stage equation; and second, the exclusion restriction needs to hold, that is, the instrument should not influence the primary outcome directly through any channel other than treatment (Mostert and Vall, 2020, Mostert, 2021, Mostert, 2021, Mostert, 2021, Mostert, 2022). In this case, this assumption means that differences in health outcomes between the treated and control groups can only be due to exposure to the 2008 pension reform.

Due to the voluntary principle of the 2008 pension reform, pension receipt may be an endogenous household decision, which may be related to unobservable household characteristics (Lemieux and Milligan, 2008). As we include the cohort (or year) fixed effects, we are capturing any improvement (or deterioration) in mental health and deaths in any subsequent cohort (with respect to the previous ones) that may be due to other causes such as policy reforms, changes in cultural habits or economic development (Mostert and Vall, 2020).

For example, in the Appendix, let us look at Fig. 3, which defines the treated and control groups. There is no reason to believe that cohorts born from 1942 to 1947 (control group) should have different health outcomes than the cohorts born from 1948 to 1954 (treatment group) when observed at the same age (60 years old), with the same income category (less than US $ 36,567 per year) and living in the same regions, after controlling for the year (or cohort) fixed effects. No other event in South African history explains any difference in mental health outcomes that would affect precisely the cohort born from 1948 to 1954 (treatment group) but not the cohort born just one, two, or three years before, from 1947 to 1942. For this reason, we are confident that the exclusion restriction is satisfied in this case. In any case, in the robustness tests section, we will provide additional exercises (placebo regressions in the Appendix) that will provide more substantial evidence to reinforce the fulfilment of the exclusion restriction assumption in our context (Mostert and Vall, 2020).

## 4 Results

### 4.1 Descriptive analysis

Table 1 presents the exact difference between the treated and control groups. We noted that 60-year-old men who benefitted from the 2008 pension reform (treated group) recorded a low probability of depression cases, traumatic stress cases, and deaths than those not treated with the 2008 pension reform (control group). The treated group also had higher healthcare utilisation than the control group.

**Table 1:** Descriptive statistics.

Variable	Treated Cohorts	Control Cohorts
Reported being married	64.3 %	62.8 %
Reported still doing odd jobs to survive	2.4 %	3.8 %
Reported owning a business	0.9 %	0.7 %
Reported completed secondary education	2.4 %	2.5 %
Reported previous occupation as blue-collar worker	81.4 %	80.8 %
Reported living in urban rich provinces	58.0 %	57.8 %
Reported healthcare utilisation	76.4 %	74.1 %
Reported depression cases	3.9 %	5.1 %
Reported traumatic stress cases	1.6 %	3.0 %
Reported 60-year-old deaths	46.9 %	49.5 %
Observations	8805	6824

Source: Own elaboration with data from General Household Survey (GHS) provided by Statistic South Africa from 2002 to 2014.

### 4.2 Results of two-way 2SLS model

The tables present how the 2008 pension reform improved the mental health of senior citizens and averted 60-year-old deaths. When analyzing the 2SLS model results, we noted that the first stage regression's F-statistic is very large, indicating the instrument's strong validity (See Table 2). Thus, in Table 2, the improved provision of pension grants proxied by the cohort instrument is a determinant for better healthcare utilisation.

**Table 2:** 2SLS estimation of the impact of 2008 pension reform on healthcare utilisation.

2SLS	60-year-old men
1st stage	Pension grant arising from the 2008 pension reform

Treatment (Birth cohorts)	0.0885***
	(0.0012)

	Healthcare utilisation
2008 pension reform	0.0229***
	(0.0051)

Year fix effect	YES
Province fix effect	YES
Race fix effect	YES
Education fix effect	YES
Mean for the dependent variable	0.7526
Observations	15,629
F-stat 1st SLS	100.2658
P-value 1st SLS	0.0004
R squared 2nd SLS	0.0511

***Denote significant p value at <0.05. Coefficients in brackets represents standard errors. Note: The results are from a 2SLS model. In the first stage equation the dependent variable is the probability of benefiting from 2008 pension reform while the instrument is a dummy variable equal to 1 for the 60-year cohorts born in 1948 to 1954 and 0 for the 60-year cohorts born in 1942 to 1947. In the second stage regression the dependent variable is a dummy variable of “healthcare utilisation”. Both regressions include year (cohort), province and race fixed effects. Source: General Household Survey (GHS) provided by Statistic South Africa from 2002 to 2014.

For example, between 2008 and 2014, healthcare utilisation improved by 2.29 % points in the cohorts who benefitted from the 2008 pension reform compared to the control. The mean for healthcare utilisation is 75.26 %. Therefore the 2008 pension reform improved healthcare utilisation by 3.04 % in the treated cohort.

The improvements in pension provision and healthcare utilisation resulted in positive spillover effects on the mental health outcomes of men. For example, the mean for traumatic stress is 2.31 %. The model shows a 0.10 % point's reduced traumatic stress cases post 2008 pension reform (See Table 3). Therefore the 2008 pension reform averted traumatic stress by 4.34 %.

**Table 3:** 2SLS estimation of the impact of 2008 pension reform on traumatic stress.

2SLS	60-year-old men
	Traumatic stress
2008 pension reform	−0.0010***
	(0.0002)

Year fix effect	YES
Province fix effect	YES
Race fix effect	YES
Education fix effect	YES
Mean for the dependent variable	0.0230
Observations	15,629
F-stat 1st SLS	102.8711
P-value 1st SLS	0.0002
R squared 2nd SLS	0.0666

***Denote significant p value at <0.05. Coefficients in brackets represents standard errors. Note: The results are from a 2SLS model. In the first stage equation the dependent variable is the probability of benefiting from 2008 pension reform while the instrument is a dummy variable equal to 1 for the 60-year cohorts born in 1948 to 1954 and 0 for the 60-year cohorts born in 1942 to 1947. In the second stage regression the dependent variable is a dummy variable of “traumatic stress”. Both regressions include year (cohort), province and race fixed effects. Source: General Household Survey (GHS) provided by Statistic South Africa from 2002 to 2014.

The model shows a 0.14 % point reduced depression cases post 2008 pension reform (See Table 4). Mean depression score is 4.53 %. Therefore, the 2008 pension reform averted depression by 3.09 %. Indeed, social pension provision reduces the risk of depression and traumatic stress and enables senior citizens to record improved mental health outcomes.

**Table 4:** 2SLS estimation of the impact of 2008 pension reform on depression.

2SLS	60-year-old men
	Depression
2008 pension reform	−0.0014***
	(0.0002)

Year fix effect	YES
Province fix effect	YES
Race fix effect	YES
Education fix effect	YES
Mean for the dependent variable	0.0453
Observations	15,629
F-stat 1st SLS	111.2284
P-value 1st SLS	0.0003
R squared 2nd SLS	0.0592

***Denote significant p value at <0.05. Coefficients in brackets represents standard errors. Note: The results are from a 2SLS model. In the first stage equation the dependent variable is the probability of benefiting from 2008 pension reform while the instrument is a dummy variable equal to 1 for the 60-year cohorts born in 1948 to 1954 and 0 for the 60-year cohorts born in 1942 to 1947. In the second stage regression the dependent variable is a dummy variable of “depression”. Both regressions include year (cohort), province and race fixed effects. Source: General Household Survey (GHS) provided by Statistic South Africa from 2002 to 2014.

We then examine whether the 2008 pension reform positively impacts 60-year-old deaths, considering that the reform positively impacts healthcare utilization and mental health outcomes. We found in Table 5 that the 2008 pension reform reduced deaths among 60-year-old men significantly. The 2008 pension reform decreases deaths among 60-year-old men by 2.56 percentage points, implying a 5.30 percent improvement in 60-year-old deaths.

**Table 5:** 2SLS estimation of the impact of 2008 pension reform on 60-year-old deaths.

2SLS	60-year-old men
	60-year-old deaths
2008 pension reform	−0.0254***
	(0.0018)

Year fix effect	YES
Province fix effect	YES
Race fix effect	YES
Education fix effect	YES
Mean for the dependent variable	0.4822
Observations	15,629
F-stat 1st SLS	102.8711
P-value 1st SLS	0.0006
R squared 2nd SLS	0.0666

***Denote significant p value at <0.05. Coefficients in brackets represents standard errors. Note: The results are from a 2SLS model. In the first stage equation the dependent variable is the probability of benefiting from 2008 pension reform while the instrument is a dummy variable equal to 1 for the 60-year cohorts born in 1948 to 1954 and 0 for the 60-year cohorts born in 1942 to 1947. In the second stage regression the dependent variable is a dummy variable of “60-year-old deaths”. Both regressions include year (cohort), province and race fixed effects. Source: General Household Survey (GHS) provided by Statistic South Africa from 2002 to 2014.

We then focus on regional-based differences in the impact of the 2008 pension reform on 60-year-old deaths. We explore the existence of heterogeneous results for rich/poor provinces. We define under-resourced provinces (rural) as those living in Eastern Cape, Free State, Limpopo, and KwaZulu Natal. We considered Gauteng, Western Cape, Northwest, Northern Cape, and Mpumalanga rich provinces. This distinction was made based on provincial GDP per capita higher than USD6.25 (World Fact, 2018). Table 6 shows that the 2008 pension reform improved 60-year-old deaths for the resourced provinces (urban population group) more than under-resourced rural regions.

**Table 6:** 2SLS estimation of the impact of 2008 pension reform on 60-year-old deaths by region.

2SLS	Rural	Urban
	60-year-old deaths	
2008 pension reform	−0.0206***	−0.0301***
	(0.0011)	(0.0014)

Year fix effect	YES	YES
Race fix effect	YES	YES
Education fix effect	YES	YES
Mean for the dependent variable	0.4786	0.4878
Observations	6773	8856
F-stat 1st SLS	88.8711	78.8945
P-value 1st SLS	0.0004	0.0005
R squared 2nd SLS	0.0566	0.0449

***Denote significant p value at <0.05. Coefficients in brackets represents standard errors. Note: The results are from a 2SLS model. In the first stage equation the dependent variable is the probability of benefiting from 2008 pension reform while the instrument is a dummy variable equal to 1 for the 60-year cohorts born in 1948 to 1954 and 0 for the 60-year cohorts born in 1942 to 1947. In the second stage regression the dependent variable is a dummy variable of “60-year-old deaths”. Both regressions include year (cohort) and race fixed effects. Source: General Household Survey (GHS) provided by Statistic South Africa from 2002 to 2014.

## 5 Discussion

Mental health has been increasingly acknowledged as a burning public health issue in South Africa (National and Strategic Plan, 2021). However, from 2002 to 2014 mental health policy was not prioritized across South African provinces (Lund et al., 2008). Hence, the current reported results cannot be attributable to past mental health policy strategies. The momentum towards Universal health coverage also indicates the need to integrated mental health policies in the overall health policy discourse.

There is emerging evidence from developing countries that mental ill-health is strongly associated with poverty and many aspects of social deprivation (Draper et al., 2009). Social pension provision is essential to avert poverty, and social deprivation can also improve the mental health of older men. In this paper, we demonstrate that early social pension payout (60 years vs 65 years) improves health outcomes for men, similar to other studies reported in the literature (Wang and Zheng, 2021, Waidler and Devereux, 2019, Pak, 2021, Sewdas et al., 2020, Oshio and Kan, 2017, McIntyre et al., 2016).

This cash transfer program is financially sustainable. For example, the World Bank shows that, in South Africa, social pensions reached a large number of disadvantaged older people in 2002 at a relatively low cost (1 % of GDP in Brazil, 1.9 % of GDP in China, and 1.4 % in South Africa). Hence, the expansion of pension provision in 2008 – which ultimately succeeded in buying improved healthcare utilization of senior male citizens and concurrently averted depression, traumatic stress, and 60-year-old deaths.

The impact of the 2008 pension reform is more robust for the urban region than rural settings, similar to the evidence reported by other authors (Mostert, 2021, Mostert, 2021), where better health outcomes were associated with individuals from the developed urban regions with better economic activities and resources. One possible reason for this outcome is that urban areas of South Africa are often densely populated with modern healthcare facilities, medical personnel, and other drug and technological resources. These health system inputs ensure the bias improvement in health outcomes of senior citizens from urban regions compared to rural populations.

### 5.1 Policy implications

Delaying pension initiation to 67 or 70 years, as popularly implemented in other countries, may not serve the health interest of senior male citizens from disadvantaged households. Our results are consistent with findings reported in Canada (Emery et al., 2013), where delayed pension provision was associated with poor health outcomes. On the other hand, early pension provision in Canada leads to significantly better mental health and improved overall health of senior citizens (McIntyre et al., 2016). In this paper, we discovered that the lack of social pension provision in South Africa is associated with low healthcare utilization, reinforcing older men's poor mental health outcomes. Therefore, those calling for delayed pension initiation may inadvertently worsen health outcomes for senior citizens, considering the existing poverty and income inequalities in the Southern African elderly population.

Our findings have critical policy implications. First, because the beneficial effects of the 2008 pension reform on the deaths of rural 60-year-old men are minimal compared to urban men--the South African government may need to increase social investment in rural areas and help the rural elderly men relieve depression and traumatic stress from poverty. Second, because pension income has some beneficial effects on the mental health of lower income 60-year-old men, further reforms of the South African social pension system are warranted. For example, the social pension system should give more subsidies to elderly persons with poor economic conditions to help raise income and improve mental wellbeing. One possible amendment which can be adopted -is having all social pension payouts pay slightly above the inflation rate to prevent the erosion of these funds from inflation dynamics.

### 5.2 Robustness checks

Finally, we ran some placebo regressions in which the study “pretended” that comparison (unaffected) cohorts were treated with fake pension reform. Thus, we excluded from the sample the cohorts that were genuinely affected by the 2008 reform. We then assigned as treated cohorts affected by the fake reform those born in 1942, 1943, and 1944 and used as comparison cohorts those born in 1945, 1946, and 1947. We then run the same 2SLS.

One can see in Table 10 in the Appendix that the F-test of the first stage regression is extremely low (which suggests that the instrument is not relevant). The treatment variable is not significant in any of the health outcomes analyzed: therefore, the results of these placebo tests analyzing the effects of the fake reforms reinforce the validity of the study's identification strategy and provide additional evidence of the fulfilment of the exclusion restriction criteria as any cohort-specific events not captured by the year effects that could be biasing the main results should also provide significant results in these placebo tests (Mostert and Vall, 2020, Mostert, 2021, Mostert, 2021, Mostert, 2021).

### 5.3 Limitations

There is no advance information on the determinates of depression and traumatic stress in the Statistics South Africa data. Thus, we interpret the results as providing evidence of substantial improvement in these outcomes attributed to the 2008 pension reform while not capturing other qualitative changes that may further explain the advancement of these outcomes. For example, elderly mental health outcomes can also be influenced by religious factors. This aspect could bias the mental health outcomes. Religious and spiritual beliefs reduce the multiple behavioral risk factors (Linardakis, 2015).

Unfortunately, the Statistics South Africa data does not contain religious information and frequency of praying, which should have been controlled in the analysis. Nevertheless, we believe such omission will not significantly influence the current estimation considering that pension provision is still a competent primary driver of better mental health outcomes for senior citizens and avert deaths.

## 6 Conclusion

The 2008 pension reform is crucial in improving male senior citizens' healthcare utilisation and mental health conditions. The reform successfully bought the mental health of male senior citizens and averted depression cases, traumatic stress cases, and deaths among 60-year-old men.

## Funding

This research did not receive any specific grant from the public, commercial, or not-for-profit funding agencies.

## CRediT authorship contribution statement

**Cyprian M. Mostert:** Conceptualization, Methodology, Writing – original draft. **Diana Mackay:** Writing – original draft. **Alex Awiti:** . **Manasi Kumar:** Visualization, Investigation, Writing – review & editing, Writing – original draft. **Zul Merali:** Supervision, Validation, Writing – review & editing.

## Declaration of Competing Interest

The authors declare that they have no known competing financial interests or personal relationships that could have appeared to influence the work reported in this paper.

## Figures and Tables

**Table 7:** OLS estimation of the impact of 2008 pension reform on depression.

OLS	60-year-old men
	Depression
2008 pension reform	−0.0033***
	(0.0004)

Year fix effect	YES
Province fix effect	YES
Race fix effect	YES
Education fix effect	YES
Mean for depression	0.0453
Observations	15,629

***Denote significant p value at <0.05. Coefficients in brackets represents standard errors.

**Table 8:** OLS estimation of the impact of 2008 pension reform on 60-year-old deaths.

OLS	60-year-old men
	60-year-old deaths
2008 pension reform	−0.0491***
	(0.0007)

Year fix effect	YES
Province fix effect	YES
Race fix effect	YES
Education fix effect	YES
Mean for 60-year-old deaths	0.4822
Observations	15,629

***Denote significant p value at <0.05. Coefficients in brackets represents standard errors.

**Table 9:** OLS estimation of the impact of 2008 pension reform on trauma.

OLS	60-year-old men
	Trauma
2008 pension reform	−0.0023***
	(0.0004)

Year fix effect	YES
Province fix effect	YES
Race fix effect	YES
Education fix effect	YES
Mean for trauma	0.0230
Observations	15,629

***Denote significant p value at <0.05. Coefficients in brackets represents standard errors.

**Table 10:** Impact of fake pension reform on mental health and physical inactivity.

2SLS	Urban	Rural
	60-year-old deaths	
Fake pension	−0.9812	−0.8812
	(0.7941)	(0.9011)

Year FE	YES	YES
Province FE	YES	YES
Race FE	YES	YES
Education FE	YES	YES
Observations	3428	3396
F-stat 1st SLS	0.4401	0.2000

	Trauma	
Fake pension	−0.7541	−0.6651
	(0.9587)	(1.0058)
Year FE	YES	YES
Province FE	YES	YES
Race FE	YES	YES
Education FE	YES	YES
Observations	3428	3396
F-stat 1st SLS	0.5155	0.1295

Coefficients in brackets represent standard errors—source: General Household Survey (GHS) provided by Statistic South Africa from 2002 to 2007.

## Data Availability

Data will be made available on request.
